# Management of primary cardiac leiomyosarcoma

**DOI:** 10.3332/ecancer.2023.1562

**Published:** 2023-06-15

**Authors:** Lucrecia Aline Cabral Formigosa, Luciana Ferreira dos Santos, Jaqueline Dantas Neres Martins, Bianca Silva de Brito, Hemilly Vasconcelos de Miranda Silva, Ricardo Luiz Saldanha da Silva, Josinete da Conceição Barros do Carmo, Samara Machado Castilho

**Affiliations:** 1 Population Based Cancer Registry of Belém, Belém 66093-677, Brazil; 2 Federal University of Pará, Belém 66075-110, Brazil; 3 Amazonia University, Belém 66060-902, Brazil

**Keywords:** leiomyosarcoma, heart atria, heart neoplasms, case report

## Abstract

**Background:**

Primary cardiac cancer is a rare event with various clinical presentations and often causes unexpected symptoms or sudden death. Case reports with this diagnosis are scarce.

**Case presentation:**

We present an unusual manifestation of leiomyosarcoma of the left atrium in a female patient, 33 years old. Presenting difficulty to walk, dyspnoea at rest, skin pallor, cough with hemoptoics and syncope. A transthoracic echocardiogram showed cavitary enlargement of the left atrium, moderate to significant mitral stenosis with an adherent mass in the anterior leaflet, left ventricular systolic function preserved at rest, and mild aortic and tricuspid insufficiency. The procedure was complete resection of the tumour or negative microscopic margins (R0 resection), 25 sessions of radiotherapy, 5 cycles of adjuvant chemotherapy using gemcitabine (900 mg/m^2^ on days 1 and 8) and docetaxel (75 mg/m^2^ on day 8), with a resolution of the clinical picture. After 5 years of follow-up, the patient had no metastases or recurrence of the initial tumour.

**Conclusion:**

The nonspecific symptoms presented in the reported case demonstrate that the cardiac tumour can mimic other cardiac disorders, such as coronary artery disease or pericarditis, rarely representing the first manifestation of a previously unknown malignancy.

## Background

Malignant heart tumours are rare and present a nonspecific clinical picture which can be confused with other heart diseases, among them: arrhythmias, acute coronary syndromes and pericarditis. They constitute about 0.25% of all cardiac neoplasms found in men and women [1, 2].

Sarcomas with cardiac topography represent 1% of the other sarcomas [3]. As a primary cardiac cancer, this morphology is the most common, with heterogeneous characteristics and the presence of infiltration in distant organs, such as lung, kidneys and liver [2].

During the work of professionals from the Population-Based Cancer Registry of Belém/Pará/Brazil, a rare case of left atrial leiomyosarcoma (LMS) was reported.

This study aims to describe a case of left atrial LMS, addressing the way it was diagnosed, treated and followed up for 5 years. As these objectives were achieved, the relevance of the study and the contributions to the medical literature on the subject were evidenced.

## Case description

A woman, 33 years old, married, merchant, 8 years of schooling, Jehovah's witness, from the countryside of the state of Pará/Brazil, attended the Gaspar Vianna Clinical Hospital Foundation in May/2015 complaining of difficulty to walk, dyspnoea at rest, cough with hemoptoics sputum and syncope.

Since the age of 16, she had dyspnoea and fatigue, with worsening of symptoms after the first pregnancy, at 17 years. In the same period, she started to experience frequent episodes of headache, palpitations and hypertension, a condition that was treated as anxiety attacks. At the age of 32, she reported loss of motor strength, drowsiness, fatigue and indisposition to daily activities.

After worsening the condition, in 2015, at the age of 33, she sought specialised care in Belém, the capital of the state of Pará, being referred to the reference institution in cardiology of the public network of the region with a clinical picture of acute pulmonary oedema with aetiology yet to be determined.

On physical examination, she demonstrated tachypnoea, skin pallor (++/+++), fine basal rales and moderate mitral systolic murmur. She had no diagnosis of primary heart disease and denied exposure to chemicals and toxic materials.

**Figure 1.**
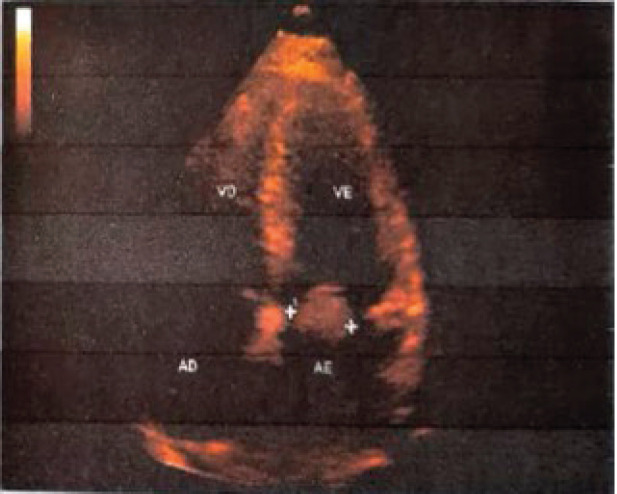
Echocardiogram with transthoracic Doppler showing a mass in the region. Source: Image provided by the patient.

She underwent transthoracic echocardiography (Figure 1), which showed cavitary enlargement of the left atrium, moderate to important mitral stenosis with mass adhered to the anterior leaflet, a systolic function of the left ventricle preserved at rest and mild aortic and tricuspid insufficiencies. Based on these findings and epidemiology, the hypothesis that it was a benign tumour called myxoma was raised.

She underwent urgent cardiac surgery at the institution for complete removal of the lesion or R0 resection of an adipose nodule encapsulated in the wall of the left atrium measuring 6.0 cm, in its largest diameter, which obstructed the mitral valve.

**Figure 2.**
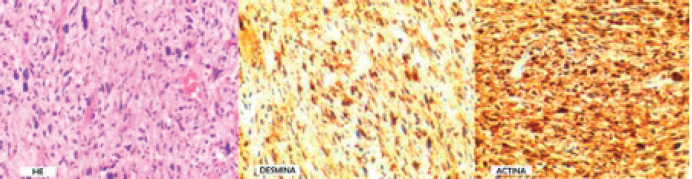
Immune-histochemistry. Source: Image provided by the patient.

Anatomopathological analysis revealed negative microscopic margins for tumour. The morphology described was myxoid fusocellular tumour with cellular anaplasia, and the immunohistochemical study with desmin (Figure 2) showed that the neoplastic cells were positive for D33 and smooth muscle actin 1–4; and negative for CD34, AE1/AE3, FSD and protein S100; findings compatible with undifferentiated high-grade LMS in the left atrium.

The young woman was subsequently referred to the oncology centre in the state of Pará/Brazil, where the initial proposal was to perform five cycles of adjuvant chemotherapy, therapy administered every 21 days, with Gemcitabine (900 mg/m^2^/day) – 1,620 mg (days 1 and 8) and Docetaxel (75 mg/m^2^/day) – 135 mg (day 8), in addition to receiving the granulocytic colony-stimulating factor (days 9–13). However, before the third cycle, she presented adverse effects, such as myalgia (grade 1), asthenia (grade 1), mucositis (grade 1), nausea (grade 1), diarrhoea (grade 2) and arthralgia (grade 2). It was necessary, from the third cycle, to increase the chemotherapy interval to 28 days and reduce the initial dose of Docetaxel by 15%, remaining 63 mg/m^2^/day or 114 mg, until the fifth cycle.

Concomitant to chemotherapy, she performed three-dimensional conformal radiation therapy, 25 sessions, in the cardiac area, with a total of 50 Gr in 25 phases of 2 Gr/day or PTV 200 cGy/day and a total of 5,000 cGy, in the period from January 4 to 2 March 2016.

After these sequences of treatments with post-surgical radiotherapy and chemotherapy, no evidence of local or systemic oncological disease was detected, proven by staging tests. However, the patient presented cardiac sequelae, with moderate symptoms of arrhythmia and heart failure, well controlled with the use of medication: Enalapril, Carvedilol and Ancoron.

In 2018, she performed a diagnostic investigation of breast and lung nodules, excluding malignant neoplastic processes.

In 2020, 5 years after diagnosis and treatment, the patient remained in complete remission, in follow-up with the multiprofessional oncology team. She used medications for systemic arterial hypertension, diabetes mellitus, heart failure and arrhythmia.

## Discussion

Cardiac neoplasms are uncommon conditions in the health service and they can be classified as benign and malignant. The latter are further divided into primary or secondary, according to the location of the formation of malignant tissue [1, 2].

Cardiac neoplasms are most often benign, and among these, myxoma stands out with the highest number of cases [1, 3, 4].

Malignant cardiac tumours often have their diagnosis for sarcoma hampered by the epidemiological relevance of myxoma, a type of benign tumour more incident among primary tumours [3, 10]. Evidence of malignancy comes from the presence of tumour invasion in adjacent tissues [9], which only occurs in advanced stages of the disease. The differential diagnosis is based on histological and immunohistochemical evaluation [3, 9]. Considering these aspects, there is a noteworthy similarity with the reported case, which obtained a diagnosis suggestive of myxoma, until the histological and immunohistochemical finding of malignancy.

Among the malignant tumours, secondary neoplasms correspond to the highest number of cases, which come from metastases and constitute more than half of the occurrences [2, 3]. On the other hand, primary cardiac cancers are rare, highly aggressive and responsible for an unfavourable prognosis [1, 3, 6, 7]. Among the latter, the various types of sarcomas (angiosarcoma, rhabdomyosarcoma and LMS) stand out, corresponding to 75% of all cardiac cancers [1, 3–5].

The most common location of cardiac malignant cancer is the left atrium and the most incident malignant tumour is angiosarcoma, with aggressive behaviour and a worse prognosis [7].

As for LMS, the main sites affected are the uterus, abdomen and retroperitoneum [11], which leads them to be divided into uterine and non-uterine LMS [2]. As cardiac tumours, they are extremely rare [7, 9]. Primary cardiac LMS are extremely rare [6, 8] and have a worse prognosis [6, 7].

In the case described here, the tumour behaved in a similar way in terms of location, but diverged regarding differentiation, as it had undifferentiated behaviour and a high degree of invasion, leading to the option for radiotherapy and adjuvant chemotherapy, despite complete resection of the lesion or R0 resection. The complete removal of the tumour contributes to the improvement of cardiovascular symptoms and survival of patients and it becomes more effective when associated with radiotherapy and adjuvant chemotherapy [8, 9].

In the patient of the case described here, the combination of chemotherapies gemcitabine (900 mg/m^2^) and docetaxel (75 mg/m^2^) was performed and good effectiveness was obtained, probably due to the fact that she had previously undergone R0 surgery. In other cases of cardiac LMS found in the literature, this combination had a modest effectiveness [9].

Cardiac sarcomas develop in adult-young individuals aged up to 40 years [6, 13], similar to what happened in the present study, in which the patient was 33 years old.

This type of neoplasm is difficult to diagnose since they are asymptomatic until they are in advanced stages, when the patient presents symptoms at the cardiovascular level that can be confused with other heart diseases [9].

Atrial neoplasms often cause nonspecific symptoms consistent with other heart diseases such as arrhythmias, cardiac insufficiency, endocarditis and pericarditis [1, 2]. The symptoms include palpitations, precordial pain and dyspnoea [9], as reported by the patient who manifested such symptoms for an extended period, in addition to problems with blood pressure control, identified as anxiety.

In addition, the diagnosis of systemic arterial hypertension at a young age, less than 30 years, is an issue to be analysed, as it is characterised as a secondary hypertensive stage, and the secondary cause should be investigated [14]. In this patient, this issue was not only left uninvestigated, but it was also confused with anxiety.

Surgery to remove the atrial neoplasm stands out in the treatment. In the case analysed, the surgery was successful, with the complete resection of the tumour. The benefit of this procedure was the reduction of the presented symptomatology, the possibility of a better management of the case with other adjuvant therapeutic modalities and a better prognosis.

The patient in the present case report continued to attend consultations during 5 years after her treatment. She was still healthy and undergoing therapy for underlying diseases, contrary to other case reports found in the literature, in which patients had a cancer-free survival of approximately 9–12 months [15].

## Conclusion

This report highlights the importance of this description. As it is a rare type of primary cancer with few findings in the literature, the present study adds to the existing knowledge. The study also draws attention to the importance of correct diagnostic elucidations.

## Author contributions

BSB, HVMS and RLSS wrote the manuscript. JDMN and JCBC reported the pathology and took part in writing the manuscript. LACF, LFS and SMC contributed to the search, draft, and analysis of data. LACF, LFS and SMC supervised the whole project. All authors read and approved the final manuscript.

## Conflicts of interest

The authors declare that they have no conflict of interest

## Funding

This research received no external funding.

## Ethics approval and consent to participate

The study was sent to the ethical committee of Gaspar Vianna Clinical Hospital, and it was approved by reference number 26901719.1.0000.0016.

## Consent for publication

Written informed consent was obtained from the patient for publication of this case report and any accompanying images. A copy of the written consent is available for review by the Editor-in-Chief of this journal.
